# Trained Innate Immunity of Fish Is a Viable Approach in Larval Aquaculture

**DOI:** 10.3389/fimmu.2019.00042

**Published:** 2019-01-25

**Authors:** Zuobing Zhang, Heng Chi, Roy A. Dalmo

**Affiliations:** ^1^School of Life Science, Shanxi University, Taiyuan, China; ^2^Key Laboratory of Experimental Marine Biology, Institute of Oceanology, Chinese Academy of Sciences, Qingdao, China; ^3^Research Group Aquaculture and Environment, Norwegian College of Fishery Science, Faculty of Biosciences, Fisheries and Economy, University of Tromsø—The Arctic University of Norway, Tromsø, Norway

**Keywords:** trained innate immunity, aquaculture, fish, beta-glucan, fish larvae

## Abstract

The general understanding has been that only adaptive immunity is capable of immunological memory, but this concept has been challenged in recent years by studies showing that innate immune systems can mount resistance to reinfection—as the innate immune system can adapt its function following an insult. Innate immune training offers an attractive approach in intensive fish larval rearing, especially since the adaptive immune system is not fully developed. Trained innate immunity will potentially favor robust fish in terms of resistance to viral and bacterial diseases. So-called immunostimulants such as ß-glucans have for decades been used both in laboratories and in intensive fish aquaculture. Treatment of fish by ß-glucans (and by other substances with pathogen-associated molecular patterns) often induces activation of non-specific/innate immune mechanisms and induces higher disease resistance. The reported effects of e.g., ß-glucans fit nicely into the concept “trained innate immunity,” but the research on fish does not yet include analysis of epigenetic changes that may be a prerequisite for long-lasting trained innate immunity. In this “perspective,” we will discuss how in practical terms and based on prior knowledge one can introduce innate immune training in brood stock fish, and their offspring, and whether innate immune training by ß-glucans is a viable approach in larval aquaculture.

## Introduction

ß-glucans are naturally occurring polysaccharides consisting of glucose residues with ß-1, 3→ß1, 4 or/and ß-1, 6→D-glycosidic bonds with various degree of polymerization. They are major structural components of the cell walls of many organisms such as fungi, plants, mushrooms, bacteria, and yeasts. ß-glucans have been reported to possess anti-cancer, pro-inflammatory, and anti-fungal activities when administered to animals. Some reports have even indicated anti-parasitic effects. Stimulation of non-specific defense mechanisms has also been reported in fish (1–4). The terminology typically used to indicate any activation of immune mechanisms is either “priming,” “immune induction” “immunostimulation” or “immunomodulation,” explaining the outcome of a particular treatment (5). More recently the terminology has more or less shifted and is now called trained immunity, or better—trained innate immunity if the effects from the particular treatment induces non-specific/heterologous disease resistance, it is relatively long-lasting and it induce epigenetic changes (5, 6). In this perspective, we will discuss how in practical terms and based on prior knowledge one can introduce innate immune training in brood stock fish, and their offspring, and whether innate immune training by ß-glucans is a viable approach in larval aquaculture.

## Trained Innate Immunity: Concept and Examples

Trained innate immunity can be explained by innate immune defense stimulation that may in turn confer increased non-specific resistance to infection by homologous or heterologous pathogens. Examples have been retrieved from research on plants and invertebrates, where a certain kind of memory from previous insults exists (7). In vertebrate species, several approaches using ligands to pattern recognition receptors (e.g., ß-glucan, muramyl dipeptide, CpG containing oligodeoxynucleotides, flagellin) have suggested that priming of mice by some of these stimulants may facilitate protection against infection by heterologous pathogens (8). An illustrative example is where pretreatment of ß-glucan coated microbeads fully protect mice against an otherwise lethal *Escherichia coli* challenge in mice (9). Other examples are the prophylactic effects from a ß-glucan (laminaran) injection against *Vibrio salmonicida* infection in fish (10) and where Bacillus Calmette Guerin (BCG) vaccination induce T-cell independent non-specific disease protection against infections of e.g., *Candida albicans* and *Schistosoma mansoni* in mice (11, 12). Thus, the term trained innate immunity is a new wrapping of what has been observed and reported decades ago. However, a few more characteristics have since been added to the concept due to more research and the use of modern technologies. These are: T- and B-cell independent process, epigenetic changes together with altered metabolic profile (13). The mechanisms behind priming or training are acknowledged to be functional (re)programming of cells (monocytes, macrophages, NK cells) induced by activation of particular pattern recognition receptors, mainly MAP kinase dependent intracellular signaling, and resulting epigenetic changes (8, 14). It should be noted that there exist other venues where cross-protection occurs. Poly-specific lymphocytes, the Mackaness reaction (chronic infection), and microbiota-mediated protection may all be venues to protective mechanisms, reviewed by Muraille (15). It is commonly acknowledged that B-cell produced antibodies, including natural antibodies, are not involved in trained innate immunity (16). Sea water is extremely rich in microbes (phages, viruses, bacteria), containing molecules that may induce immune activation or tolerance. The fish gut microbiome has been reported to consist of many species of the proteobacterial phylum (17). These are gram-negative bacteria with bacterial lipopolysaccharide (LPS) in their outer membrane, which is known to induce substantial immune activation. An attractive research question is why continuous exposure of high amounts of environmental LPS does not induce hyperactivation of the immune system. This issue may be dependent on the dose, where sensitization occurs by a low-dose LPS, whereas priming with high-dose LPS induces prolonged inhibition of inflammatory cytokine release—dependent on the mTOR (mammalian target of rapamycin) and AMPK (AMP-activated kinase) signaling axes. Such inhibition can be translated as LPS tolerance (8, 18, 19). mTOR is involved in anabolic processes during cell activation, whereas the latter is central in tissue homeostasis and tolerogenic responses. It is not yet clear whether ß-glucans themselves induce tolerance that would be detrimental to their stimulating effects. Moreover, many fish species possess several splicing isoforms of e.g., PPRs where an activation of one of the spliced isoforms of a given pattern recognition receptor (12) might give another outcome (e.g., negative regulation) than expected (20–22).

## ß-glucans: Not All Are Alike

Since there are high level of heterogeneities (and impurities) among different commercial preparations of ß-glucans from various sources cautions must be made (23). One type of ß-glucan from one species can be very different with respect to solubility in e.g., PBS/saline and gelling characteristics, compared to another ß-glucan preparation. Zymosan (*Saccharomyces cerevisiae*), the most widely applied and investigated ß-glucan, is composed of ~50% ß-glucan, 17% mannan, 14% protein, and other substances (24, 25). Zymosan (average 3 μm particles) is extremely aqueous insoluble, but the particles can be dispersed in solutions. Other well-studied biologically active ß-glucans includes laminarin, curdlan, lentinan, scleroglucan, and schizophyllan. In many cases their names are trivial describing their sources; Lentinan from *Lentinula edodes*, scleroglucan from *Sclerotium* sp., and schizophyllan from *Schizophyllum commune* (26). These microbial or fungal ß-glucans possess various degrees of polymerization that dictate, in some instances, a higher order of conformation—they are either linear and unbranched, or branched with single glucose residue—which in turns determines aqueous solubility, gelling characteristics, and often biological activities (27). Many ß-glucans have been reported to possess biological activities, such as induction of disease resistance, in both animals (vertebrates, invertebrates) and plants (4, 28–30).

## Which ß-glucans Induce Trained Immunity?

It is suggested that in order to induce trained innate immunity by ß-glucans, several different receptors must be engaged, such as Dectin-1, and dimeric TLR2/6 (31). The simultaneous binding of ß-glucan to two or more different receptors in clusters normally gives a higher response, compared to a single receptor. It is acknowledged that, among ß-glucans, particulate ß-glucans may be the optimal preparation to induce innate immune training, whereas low molecular weight ß-glucans (e.g., laminarin) do not favor a high response (32).

## High Diversity of Innate Receptors for Innate Training

Teleost fish constitute a highly diverse group of animals, comprising of more than 23,000 different species. Twenty-one different Toll-like receptors (TLRs), together with additional splicing variants (subtypes/isotypes), have thus far been identified in teleost, reviewed by Chang et al. (22) and Nie et al. (33). The number of TLR variants far exceeds that found in mice and human (34). Furthermore, a recent analysis of the Atlantic cod genome and RNAseq analysis revealed that the cod TLR repertoire is extremely diversified, with 43 different TLRs ortologs and paralogs (35). Another example is that of the blue-spotted (*Periophthalmodon schlosseri*) and giant-fin (*Periophthalmus magnuspinnatus*) mudskipper genomes which contain 11 copies of TLR13 (36). Genome duplication events in fishes during evolution has been attributed to the diversity of TLRs, thus differences with respect to the number of TLR loci exist between mammalian species and many fish species (34). The number of TLRs added to other pattern recognition receptors (12) (including splice variants) such as different C-type lectin receptors, NOD-like (nucleotide-binding oligomerization domain-like) receptors (NLRs), RIG-1-like receptors, and scavenger receptors (37), suggests that fish may very well be equipped with innate receptors that may likely be targets for innate immune training. Especially NLRs has been found to be highly expanded as shown in zebrafish, where nearly 400 NLR proteins are encoded in the zebrafish genome (38). The TLRs and NLRs outnumbers RIG-1-like receptors (39) and scavenger receptors (40) in fish, but future genomic analysis may reveal whether there are more copies of the two latter receptor families. The NLRs may likely be involved in gut responses to microbiota, as NOD1/2 are expressed on gut (zebrafish) epithelial cells (41).

One may strongly assume that trained innate immunity also exists in fish, but no definite proof exists—especially with regards to both epigenetic and metabolic changes together with the possibility of rewiring the trained state. Suggestions that trained innate immunity indeed is present in fish are based on experiments using ß-glucans and other immunostimulants *in vitro* and *in vivo*, summarized by Petit and Wiegertjes (4) and Rojo-Cebreros et al. (42).

## Trained Innate Immunity in Brood Stock Fish and Fish Larval Rearing

Given that it is possible enhance the innate defense of fish through immune training—especially against pathogens—it opens up for several interesting approaches in fish larval rearing. Firstly, brood stock (female and male) fish may be stimulated with PAMP(s) at a low dose (16) inducing increased potential to, not only resist present pathogens, but to also transfer trained innate immunity to offspring (F1 generation). This is in line with a study by Beemelmanns and Roth that suggested the occurrence of maternal and paternal transfer of immune traits. In this study they found that pipefish (*Syngnathus tyhple*) offspring expression patterns of immune genes and epigenetic regulation is correlated to parental gene expression patterns (43). Intergenerational (F0F1) transfer of trained innate immunity has been reported for other animal species such as Artemia, oyster, red flour beetle, and humans (44–47). Interestingly transgenerational immune priming beyond F1 generation (F0F2) has also been observed in fish (*S. typhle*) (48).

Besides direct innate immune training of brood stock fish and transfer to F1 (and maybe F2) generations, molecules that are known to induce trained innate immunity may be maternally transferred and taken up by developing oocytes during vitellogenesis (49)—potentially increasing the innate defense of developing embryo/larvae—while the fish embryo or larvae is still in the eggs. The latter do not represent heritable trained traits, merely a direct innate immune stimulation of offspring. In summary, by administering immunostimulants (e.g., certain PAMPs) to brood stock fish one may obtain: (1) Direct maternal and paternal immunostimulation/training, (2) consequently inherited trained innate immunity, and (3) direct immune stimulation and training of developing embryo/larvae (inside egg chorion).

**Figure 1:**
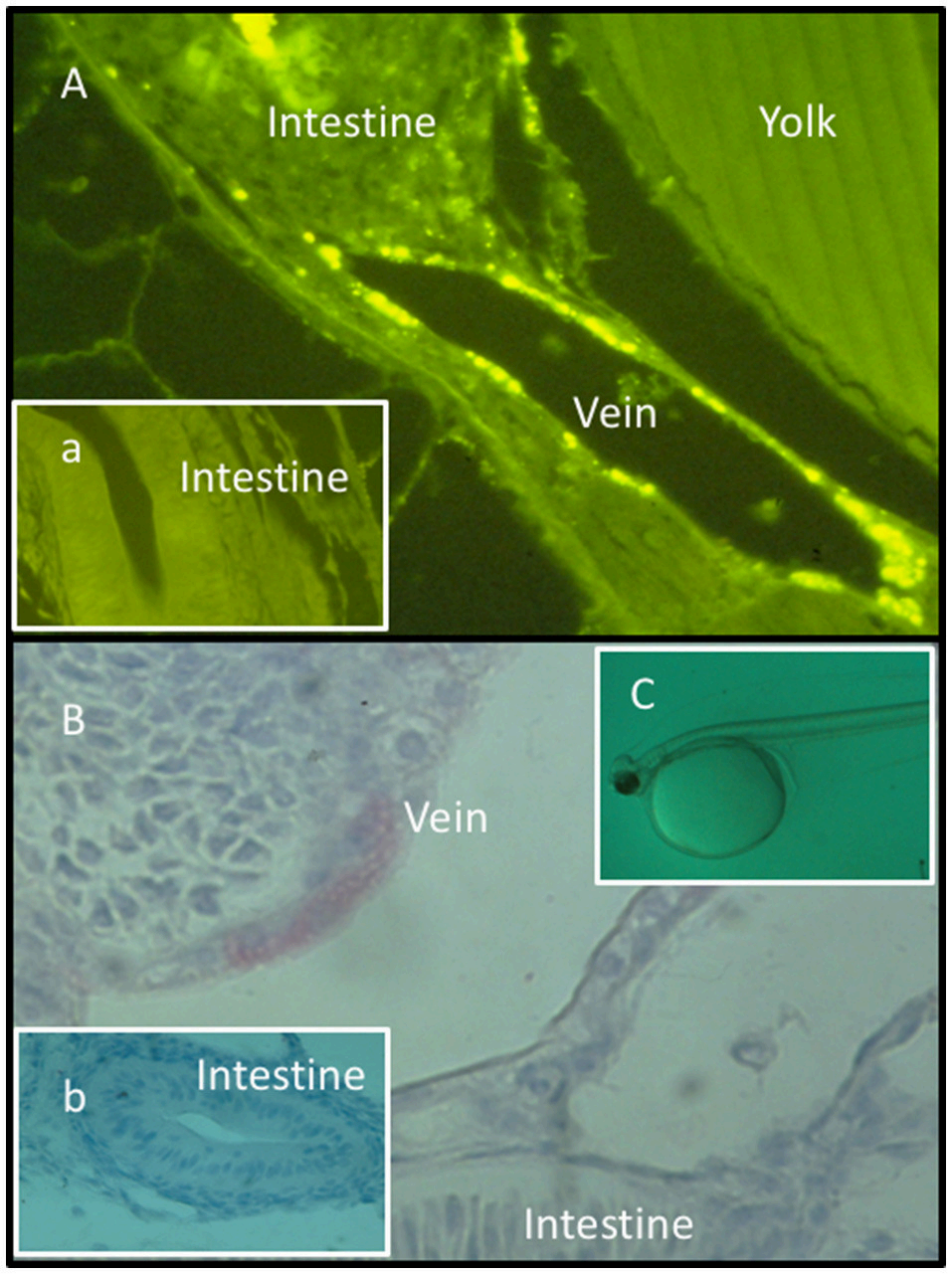
Fluorescence **(A, a)** and light micrographs (immunohistochemistry) **(B, b)** of sections from Atlantic halibut yolk sac larvae **(C)** bath treated with FITC-labeled lipopolysaccharide (FITC-LPS). **(a,b)** Sections obtained from untreated larvae. FITC-LPS was found in endothelial cells in blood veins and in intestinal tissue documenting that yolk sac larvae cells have taken up FITC-LPS after bath treatment. Permission has been granted from Elsevier for **(A)**, where the rabbit anti *A. salmonicida* pabs was characterized and used (52).

Secondly, substances expected to induce trained innate immunity may be administered directly to newly hatched fragile fish larvae or alevins (before first feeding) simply by bath treatment (50–54) (Figure 1).

Thirdly, first feeding represents a milestone during development of fish. After the yolk has more or less been utilized, the fish start feeding on algae, zooplankton, other prey, or simply pelleted formulated fish feed [for overview see Davies (53)] (55). For those fish species that feed on particulate feed, immunostimulants may simply be added to the formulated fish feed—for the purpose to induce innate immune training or immunostimulation (42, 50, 53).

## Potential Negative Effects of Early Trained Innate Immunity

If the initial stimulation, with purpose to induce innate immune training, otherwise induces hyperimmune responses in the mother/father or offspring it may give unwanted effects (56, 57)—especially in vulnerable offspring that have not fully developed regulatory mechanisms. This issue, or related issues where brood stock fish has been (over)stimulated, has not been addressed yet. An important step will be to optimize the dose and duration for full innate immune training in brood stock fish. Early trained innate immunity may, at a later time point, interfere with subsequent vaccination regime, e.g., in commercial salmonid aquaculture. The fish vaccines often contain different inactivated bacteria emulsified in mineral oil, containing many substances (58) that potentially have effect on innate defense mechanisms. Would the trained characteristics in non-vaccinated individuals be wiped out/rewired or further potentiated? One should also address whether innate trained immunity affects (later) antibody response from vaccination, especially since there is an interplay between innate pattern recognition receptors and acquired immunity (59).

## Conclusion

Training of innate immunity offers an interesting and attractive approach to increase disease resistance of brood stock fish, newly hatched fish larvae, and first feeding fish. Several TLR receptor ligands may be used to study innate training, assessed by modern technologies such as transcriptomics, epigenetics, proteomics, and metabolomics. In addition, *in vivo* pathogen challenge would be necessary to analyze whether a trained innate immunity has occurred or not.

## Author Contributions

All authors listed have made a substantial, direct and intellectual contribution to the work, and approved it for publication.

### Conflict of Interest Statement

The authors declare that the research was conducted in the absence of any commercial or financial relationships that could be construed as a potential conflict of interest.
